# Case report: Bilateral parotid abscess in a 54-day-old infant

**DOI:** 10.3389/fped.2023.1179560

**Published:** 2023-06-23

**Authors:** Lilin Huang, Xiaole Yang, Shumei Peng, Ronghan Li

**Affiliations:** Department of Pediatrics, Guangdong Women and Children Hospital, Guangzhou, China

**Keywords:** parotid abscess, lymphadenitis, *Staphylococcus aureus*, surgical incision, infant

## Abstract

Acute parotid abscess (PA) is rare in children and is prone to occur in neonates or preterm infants with high-risk factors. Sporadic cases of unilateral PA have been reported in older children. Here, we report a case of a 54-day-old child who developed bilateral PA due to *Staphylococcus aureus* infection. The infant showed bilateral cervical lymphadenopathy initially following a 13-valent pneumococcal conjugate vaccine (PCV13). However, bilateral PA developed 6 h after he was diagnosed with lymphadenitis on Day 9 of illness. Rapid PA progression from cervical lymphadenitis is rare. He recovered quickly under treatment with appropriate antibiotics based on susceptibility testing and surgical incision and drainage.

## Background

Acute parotitis in children is commonly caused by viral infections, such as mumps virus, Coxsackie virus, Epstein‒Barr virus, and influenza virus (1, 2). Less common causes include bacterial infection and autoimmune diseases (3, 4). Pathogens might ascend from the oral cavity along Stenson's duct, causing parotid infection. Viral parotitis is usually a self-limited illness. Bacterial parotitis is uncommon in children and can progress to acute suppurative parotitis (ASP) and even parotid abscess (PA) (5). PA can be life-threatening since it might spread to adjacent tissue, causing necrotizing fasciitis, mediastinitis, sepsis, and meningitis (6). PA usually occurs in neonates and premature infants and is rare in children (6). The high-risk group might share some features, such as prematurity, low birth weight, dehydration, prolonged orogastric feeding, immunosuppression, and parotid duct anomalies (3). In addition, contaminated breast milk or formula can also cause parotid infection. To date, several children who are older than 6 months of age with PA have been reported (5). Unilateral PA was found in the majority of previous cases (7). Immunocompetent infants younger than 3 months old with bilateral PA have been rarely reported. In this article, we present a case of a 54-day-old infant who developed bilateral PA due to *Staphylococcus aureus* (*S. aureus*) infection. Speculation of the potential mechanism of PA in infants is also discussed.

## Case report

**Figure 1:**
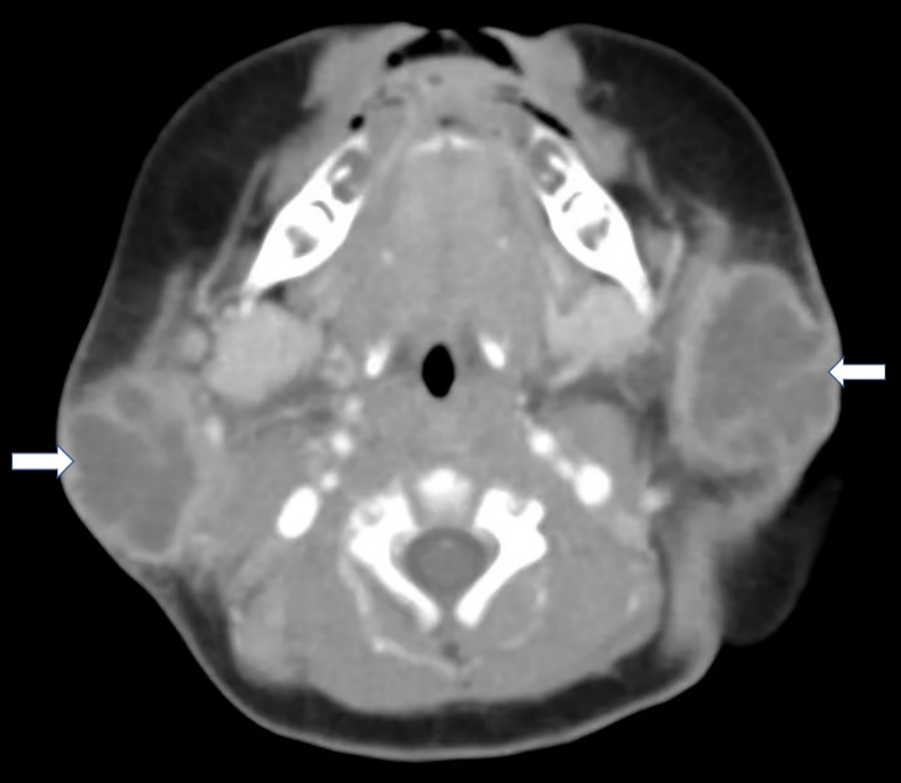
Axial contrast-enhanced CT found multiloculated low attenuation masses with rim enhancement in the bilateral parotid gland (white arrow).

A 54-day-old infant developed a mild fever (37.6°C) and bilateral neck masses the day after he received a 13-valent pneumococcal conjugate vaccine (PCV13). His fever spontaneously resolved 3 days later, and the infant was well. On Day 9 of the illness, he was brought to the first hospital due to the persistence of neck masses. Ultrasonography (USG) showed cervical lymphadenopathy with sizes of 5 mm × 4 mm (right side) and 3 mm × 5 mm (left side). He was brought home without any treatment. However, 6 h later, he showed symptoms of irritability and had a recurrent mild fever (37.5°C), along with associated preauricular swelling with erythema and fluctuation. Therefore, he was admitted to our hospital. He was born by vaginal delivery at 37^+1^ weeks of gestation, and his birth weight was 3,100 g. He was formula-fed and had no other significant prenatal or postnatal history. On admission, he was irritable, with a heart rate of 170 beats/min and a body temperature of 37.6°C. His body weight was 5,000 g (90th–97th percentile). Examination revealed bilateral fluctuated preauricular swelling approximately 4 cm × 5 cm in size with cervical lymphadenopathy. When the enlarged glands were compressed externally, pus drainage from the bilateral Stenson's duct into the mouth occurred. Laboratory tests revealed white blood cells (WBCs), 23.14 × 10^9^/L; neutrophils, 58.9%; hemoglobin, 85 g/L; platelets, 471 × 10^9^/L; C-reactive protein (CRP), 15.23 mg/L; and procalcitonin (PCT), 0.09 ng/ml. Biochemical parameters were normal. Repeated USG showed enlarged bilateral parotid glands with mixed echoic areas with several reactive lymph nodes around the gland and neck area, which were consistent with PA. A contrast-enhanced CT scan confirmed bilateral parotid abscesses with sizes of 31.4 mm × 26.3 mm × 27.3 mm (right side) and 31.1 mm × 23.2 mm × 30.5 mm (left side) (Figure 1). Cervical and submaxillary lymphadenopathy were observed. Rehydration with fluid therapy and antibiotic piperacillin–sulbactam treatment were started immediately after blood cultures were sent. Surgical incision and drainage were performed on the bilateral PA 10 h after admission. On the 3rd day of treatment, he was afebrile, and the abscesses had disappeared. Methicillin-sensitive *S. aureus* (MSSA) was detected in the pus culture from the right PA. Blood cultures were negative. Piperacillin–sulbactam was continued until the patient was discharged on the 10th day of hospitalization. He remained clinically well without recurrent parotitis on follow-up at 6 months.

## Discussion

ASP is unusual in children but is concerning since it can develop into fatal PA (5). Infants with PA who are under 3 months old have been rarely reported. We reported a case of a 54-day-old infant with bilateral PA without obvious risk factors. The typical symptoms of PA include painful parotid gland swelling with erythema and fluctuation. Pus drainage from Stenson's duct might be found (5). Pathogens might be detected in the pus culture. Other non-specific symptoms include fever, irritability, and poor feeding. On laboratory tests, some findings might include increased acute phase reactants and WBC with neutrophilic predominance. USG/CT can detect abscess formation in the parotid gland. The diagnosis is easy to establish when the patient shows typical symptoms. However, our patient developed bilateral PA 6 h after he was diagnosed with cervical lymphadenitis on Day 9 of illness. This might be related to the following reasons. First, the infant was 54 days old and was born at term. There was no evidence of feeding contaminated formula. His immune function was normal. It seemed that there were no predisposing factors for him to develop PA. Second, the infant showed a mild fever and cervical lymphadenopathy the day after he received the PCV13. The fever resolved spontaneously, and the infant was well. Therefore, the mild response to the PCV13 was speculated. Cervical lymphadenitis was diagnosed after USG detection by the emergency department in the first hospital on Day 9 of illness. However, bilateral PA developed 6 h after he was diagnosed with lymphadenitis. This indicated that PA could develop rapidly in infants, which might be spread by an infection of the peri-parotid lymph nodes. A systemic inflammatory response (SIR) was not triggered in our case. A study demonstrated that patients might be afebrile and present with slightly increased WBC levels (6). Repeated imaging detection is important for a timely diagnosis of PA if a patient shows infections of adjacent structures or deep in the neck without clinical improvement in 3–5 days or with facial nerve palsy (6). A fluctuant swelling means an abscess formation. SIR might indicate sepsis development. The interval between the two USG examinations was 6 h, but PA was formed in our case. Third, the infant showed bilateral cervical lymphadenopathy initially but not preauricular swelling, which is not a typical presentation of ASP. Rapid development of PA from an infection of neck lymph nodes is rare. In addition, the incidence of bilateral PA was lower than that of unilateral PA in previous reports. These factors brought difficulties for the early diagnosis of PA in our case.

The pathogenesis of PA is undetermined. The oral cavity is considered a source of these pathogens, which might migrate to the peri-parotid lymph node and parotid *via* the parotid duct (5). Contaminated breast milk or formula could also be the source of pathogens (8). Decreased salivary flow, poor oral hygiene, parotid duct abnormalities, and septicemia may contribute to the development of parotid infection in children (3). Our patient was born at term, and abnormality of the parotid duct was not detected. There was no evidence of the patient consuming contaminated formula or the development of sepsis. However, the infant showed mild fever for 3 days after receiving PCV13. Poor feeding and decreased salivary flow might occur during this period, which might promote bacterial ascension to the parotid gland. A study reported that a 33-day-old infant developed ASP without any risk factors (9). The infant showed unilateral preauricular swelling, irritability, and poor sucking. He did not develop PA due to obtaining a timely diagnosis and treatment with antibiotics. Therefore, there might be some undetermined factors related to PA.

*S. aureus* was the most common pathogen responsible for PA in previous cases (3). Other pathogens, such as gram-positive cocci and gram-negative microorganisms, can cause ASP. *Streptococcus pneumoniae* is also a common pathogen causing PA in children (10). Our patient showed mild fever and neck masses after he received PCV13. There might be a chance for bloodstream bacterial infection or vaccine-associated systemic inflammation that induced parotitis. However, his blood culture was negative, and his organ function was good. Pus culture indicated *S. aureus*, which is consistent with previous cases of PA.

Differential diagnosis of PA might be extensive and can include lymphadenitis, parotid gland duct anomalies, and neoplasia (9). Imaging tests are helpful to distinguish these diseases. USG detected parotid gland enlargement and hypoechoic areas in the gland, indicating suppurative parotitis. CT is more advantageous in the detection of parotid gland duct anomalies, neoplasia, and deep-neck abscesses than USG (6). In our patient, USG and CT showed enlarged parotid glands with abscesses, and reactive lymph nodes were detected in the neighborhood. No causative parotid duct-blocking diseases or complications of PA were detected by CT.

Timely treatment with fluid therapy and empirical antibiotic coverage for possible pathogens is important for ASP, and the prognosis is usually good. MSSA is usually susceptible to ampicillin and piperacillin. Vancomycin is recommended for methicillin-resistant *S. aureus*. Surgical incision and drainage might be required to control the infection if PA develops. Fatal complications such as sepsis, mediastinitis, meningitis, and deep-neck abscesses might occur if the infection progresses (6). Other complications, such as fistula and facial paralysis, might occur occasionally (10, 11). Our patient recovered quickly without any complications under treatment with appropriate antibiotics based on susceptibility testing and surgical incision and drainage.

There were two main limitations in our study. First, a pus culture from the left PA was not obtained in this patient by mistake. However, the pus culture from the right PA indicated *S. aureus*. On laboratory examinations, increased WBC and CRP were found, which also indicated bacterial infection. Second, the underlying factor that drove bilateral PA development was not found in our patient, which might cause recurrent parotid infection. Therefore, it is essential to keep monitoring this infant with follow-up.

In conclusion, we report a rare case of bilateral PA in a 54-day-old infant. PA is uncommon and might suddenly develop in infants. Pediatricians need to be alert for PA formation if infants show neck masses with fever and poor feeding in the emergency department. Early diagnosis and timely treatment of ASP with antibiotics and rehydration might help to avoid PA development. This rare case might extend the knowledge of PA in infants.

## Data Availability

The raw data supporting the conclusions of this article will be made available by the authors, without undue reservation.
